# Adult Stromal Cells Derived from Human Adipose Tissue Provoke Pancreatic Cancer Cell Death both *In Vitro* and *In Vivo*

**DOI:** 10.1371/journal.pone.0006278

**Published:** 2009-07-17

**Authors:** Beatrice Cousin, Emmanuel Ravet, Sandrine Poglio, Fabienne De Toni, Mélanie Bertuzzi, Hubert Lulka, Ismahane Touil, Mireille André, Jean-Louis Grolleau, Jean-Marie Péron, Jean-Pierre Chavoin, Philippe Bourin, Luc Pénicaud, Louis Casteilla, Louis Buscail, Pierre Cordelier

**Affiliations:** 1 Université de Toulouse, UPS, UMR 5241 Métabolisme, Plasticité et Mitochondrie, BP 84225, Toulouse, France; 2 CNRS, UMR 5241 Métabolisme, Plasticité et Mitochondrie, BP 84225, Toulouse, France; 3 Institut National de la Santé et de la Recherche Médicale U858, I2MR and IFR BMT-150, BP 84225, Toulouse, France; 4 Department of Plastic Surgery, Centre Hospitalier Universitaire Rangueil, BP 84225, Toulouse, France; 5 EFS-PM, Laboratoire de thérapie cellulaire, Toulouse, France; The University of Hong Kong, Hong Kong

## Abstract

**Background:**

Normal tissue homeostasis is maintained by dynamic interactions between epithelial cells and their microenvironment. Disrupting this homeostasis can induce aberrant cell proliferation, adhesion, function and migration that might promote malignant behavior. Indeed, aberrant stromal-epithelial interactions contribute to pancreatic ductal adenocarcinoma (PDAC) spread and metastasis, and this raises the possibility that novel stroma-targeted therapies represent additional approaches for combating this malignant disease. The aim of the present study was to determine the effect of human stromal cells derived from adipose tissue (ADSC) on pancreatic tumor cell proliferation.

**Principal Findings:**

Co-culturing pancreatic tumor cells with ADSC and ADSC-conditioned medium sampled from different donors inhibited cancer cell viability and proliferation. ADSC-mediated inhibitory effect was further extended to other epithelial cancer-derived cell lines (liver, colon, prostate). ADSC conditioned medium induced cancer cell necrosis following G1-phase arrest, without evidence of apoptosis. *In vivo*, a single intra-tumoral injection of ADSC in a model of pancreatic adenocarcinoma induced a strong and long-lasting inhibition of tumor growth.

**Conclusion:**

These data indicate that ADSC strongly inhibit PDAC proliferation, both *in vitro* and *in vivo* and induce tumor cell death by altering cell cycle progression. Therefore, ADSC may constitute a potential cell-based therapeutic alternative for the treatment of PDAC for which no effective cure is available.

## Introduction

Normal tissue homeostasis is maintained by dynamic interactions between epithelial cells and their microenvironment. The microenvironment consists of extracellular matrix, stromal cells, migratory immune cells and neural elements supported by a vascular network, all within a milieu of cytokines and growth factors. Cells interact with the microenvironment via complex autocrine and paracrine mechanisms. Multiple evidences show that disrupting this homeostasis can induce aberrant cell proliferation, adhesion, function and migration that might promote malignant behavior [1]–[3]. Recent studies have altered the perception of the stromal cells surrounding epithelial tumors. These cells are not idle bystanders, but rather active participants that shape the frequency and features of the tumors. In addition, cancer cells themselves can alter their adjacent stroma to form a permissive and supportive environment for tumor progression [1]–[3].

Cancer stem cells and stromal fibroblasts are extensively demonstrated to support neoplastic process [4]–[11], when bone marrow derived mesenchymal stem cells (BM-MSCs) indirectly favor tumor growth following systemic immunosuppression [12], or production of pro-angiogenic cytokines [13]–[15]. However, the effects of BM-MSCs on tumor proliferation vary according to the tumor type: BM-MSCs promote breast, melanoma and colon cancer derived cells proliferation [16], [17], when inhibit the *in vivo* growth of Kaposi's sarcoma cells [18]. Adipose tissue, like bone marrow, contains stromal cells called ADSCs for Adipose-derived Stromal Cells. This population shares many of the characteristics of the BM-MSCs, including immunomodulatory effects [19], and multilineage differentiation potentials [20]–[29]. Once injected *in vivo*, ADSCs exhibit regenerative properties either by direct participation in newly formed tissues and/or via production of growth factors that sustain this regeneration [23], [25], [30]. Although stromal cells from bone marrow and adipose tissue seem to be closely related, notable differences were reported [19], [31]–[34]. The ability of ADSCs to support normal and tumor cell proliferation is largely debated. *In vitro*, expanded ADSCs mediated suppression of allogeneic lymphocyte proliferation [35]. Contradictory results were obtained *in vivo*, when co-injection of ADSCs with breast, colon, prostate, non-small lung or glioblastoma cancer-derived cells led to initial tumor growth support [36]–[38]. To clarify such discrepancy, we studied the effect of ADSCs on pancreatic ductal adenocarcinoma-derived cells. Among solid tumors, pancreatic ductal adenocarcinoma (PDAC) is characterized by its extremely dense desmoplastic infiltration [39], [40]. PDAC is a highly aggressive disease with no therapeutic solution, and still the fifth leading cause of cancer-related deaths in Western countries [41]. Because aberrant stromal-epithelial interactions contribute to PDAC spread and metastasis, we hypothesize that targeting the tumor stroma may represent an additional approach for treating PDAC. Here, we demonstrate the ability of human ADSCs (hADSCs) to reduce PDAC-derived cancer cells growth, *in vitro* and *in vivo*, and to induce cancer cell death following G1-phase arrest.

## Results

### Human ADSCs exert an inhibitory effect on PDAC-derived cell lines

**Figure 1:**
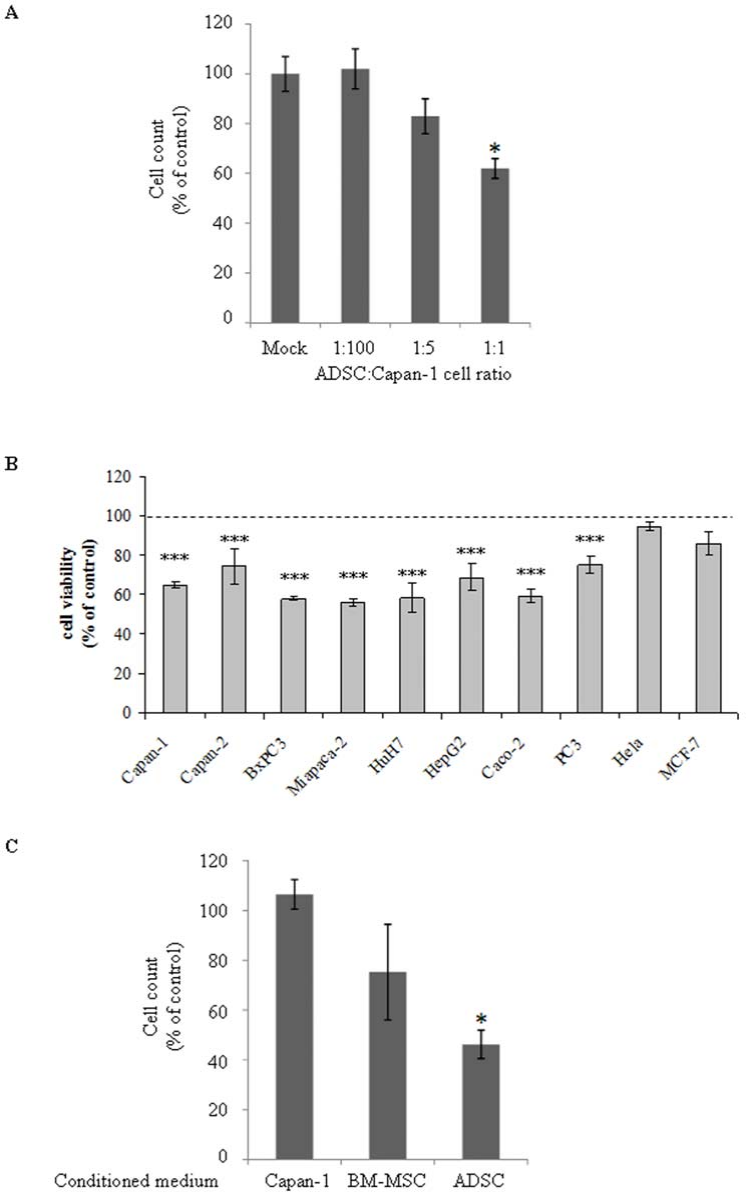
Human ADSCs induce a contact-independent inhibition of tumor cells growth and viability. A. Increasing ratios of ADSC and Capan-1 cells were co-cultured in the presence of transwell for 48 hours in full growth media. Capan-1 cells proliferation was then quantified by cell counting. Values are means±S.E. of three separate experiments with ADSCs from different donors. B. Cancer cells from diverse origins were cultured in the presence of ADSC conditioned medium (CM) for 48 hours. Cell viability was ascertained by MTS using The CellTiter 96® AQueous One Solution Cell Proliferation Assay. Results are expressed as percentage of values obtained in control conditions. Values are means±S.E. of three separate experiments performed with ADSC-CM from different donors. C. Capan-1 cells were cultured for 48 h with Capan-1, BM-MSC or ADSC-CM. Capan-1 cell number was quantified as in A. Values are means±S.E. of three separate experiments with ADSCs and MSCs from different donors. *: p<0.05. ***; p<0.001.

First, hADSC phenotype was determined by flow cytometry (Supplementary Text S1) in order to confirm that the cells used displayed phenotypic characteristics usually described for ADSCs [22], [23]. Indeed, FACS analyses confirmed that ADSC were CD34, CD90, CD73 and HLA ABC positive whereas CD45 and CD31 negative (Supplementary Figure S1). We then determined the *in vitro* effect of hADSCs on PDAC tumor cell growth. We cultured PDAC-derived Capan-1 cells in the presence of ADSCs isolated from 25 different healthy donors using transwell membranes to prevent cell-to-cell contact. After 48 hours of co-cultures, ADSC exhibited a potent dose-dependent inhibitory effect on PDAC-derived cell number, as demonstrated in figure 1A. Indeed, the number of pancreatic cancer cells was significantly decreased by 36.5%±4.8% in the presence of 1∶1 ratio of ADSCs, when compared to the control.

### Conditioned medium from ADSCs inhibits the viability of many tumor cells

The absence of direct cell–to-cell contact in our experimental setting prompted us to test whether ADSCs-conditioned medium (CM) may also impair cancer-cell viability. Indeed, soluble factors have been previously demonstrated to mediate the immunosuppressive effect of human ADSCs [19]. As shown in figure 1B, ADSC-CM by itself significantly inhibited not only the viability of several pancreatic adenocarcinoma-derived cells (Capan-1, Capan-2, BxPC3, Miapaca-2), but also the viability of hepatocarcinoma-derived cells (HuH7, HepG2), of colon-cancer-derived cells (Caco-2), and of prostate cancer cell line (PC3). In contrast, ADSC culture supernatant had little, if any, effect on Hela cells derived from cervical cancer or MCF-7, a breast cancer cell line. Because, the observed responses may reflect depletion of nutrients from media and/or non specific accumulation of toxic metabolites, we used conditioned medium from cancer cells alone as a control, together with BM-MSC-CM. In contrast to ADSC-CM, treating cancer cells with either Capan-1 or BM-MSC-CM did not impair significantly cancer cell viability although a trend of decrease was observed with the last one (figure 1C).

### ADSC-conditioned medium inhibits tumor cell proliferation and induces tumor cell death

**Figure 2:**
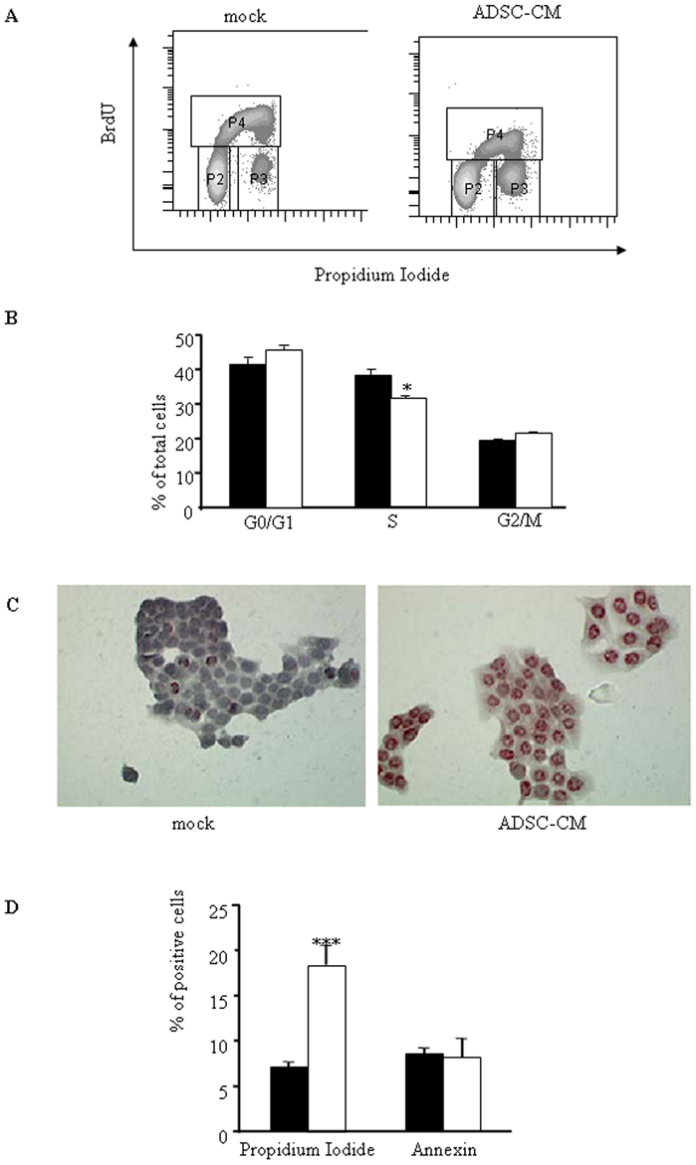
Human ADSCs inhibit cell proliferation and induce cell death in pancreatic cancer cells. A. Capan-1 cells were cultured with or without ADSCs conditioned medium was added. 48 hours later, proliferation and DNA content were analyzed by flow cytometry using BrdU incorporation and propidium iodide as described in Material and Methods. Dot plots are representative of 3 independent experiments performed with ADSCs sampled from different donors. B. Quantification of cell-cycle analysis using ModFit software. Capan cultured in control conditions were represented in black bars in contrast to Capan treated with ADSC-CM represented in open bars. Values are means±S.E. of three separate experiments with ADSCs from different donors. C. Capan-1 cells were seeded in 4-chamber slides for 48 h with or without ADSC-CM. DNA fragmentation was measured by TUNEL (representative of three separate experiments). D. Capan-1 cultured in control medium (black bars) or treated with ADSC supernatants (open bars) were assayed for Annexin and propidium iodide labelling as described in Material and Methods. Values are means±S.E. of three separate experiments with ADSCs from different donors. ***: p<0.001.

To analyze potential effect of ADSC-CM on cancer cell proliferation, Capan-1 were incubated in presence of BrdU with or without ADSC-CM. FACS analysis was performed after cell denaturation and incubation with propidium iodide. BrdU is incorporated during S phase (gate P4) whereas staining with propidium iodide allows discrimination between 2n (G0/G1, gate P2) and 4n (G2/M, gate P3) chromosomes (Figure 2A). Dot plots gated on single cells showed that the number of Capan-1 cells in G0/G1 phase tended to increase when cells in S phase significantly decreased by 10% in the presence of ADSC-CM (figure 2B).

We next determine whether ADSC-CM could affect cell death. To this end, we first monitored DNA fragmentation in Capan-1 cells by TUNEL assay. As shown in figure 2C, PDAC-derived cell DNA fragmentation was strongly induced by ADSC-CM. We next quantified the number of cancer cells entering apoptosis and/or necrotic following exposure to ADSC-CM. Results presented figure 2D demonstrate that ADSC-CM induced a 3-fold increase in propidium iodide-positive, necrotic cells, without evidence of early apoptosis monitored by annexin labeling. Again, CM from BM-MSC was ineffective in altering significantly tumor cell viability (data not shown).

### Tumor cell death induced by ADSC conditioned medium is mediated by inhibition of cell cycle progression

**Figure 3:**
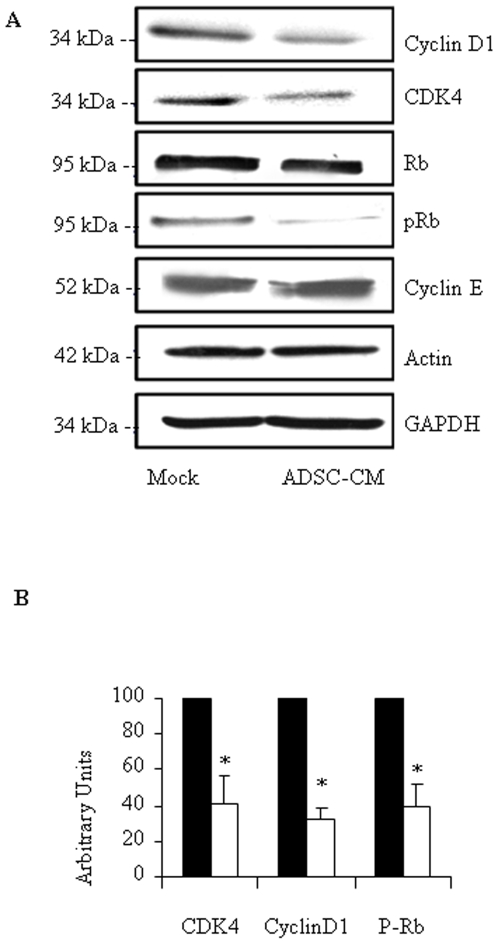
Mechanism of tumor cell cycle arrest by ADSC-CM. A. Capan-1 cells were cultured with or without ADSC-CMfor 48 hours. Proteins were then extracted and subjected to immunoblotting for the indicated proteins as described in Material and Methods. Results are representative of at least 3 independent experiments. B. Quantification of immunoblot densitometry in control cultures (black bars) or cultures treated with ADSC-CM (open bars). Values are means±S.E. of three separate experiments with ADSCs from different donors. *: p<0.05; **: p<0.01.

We next investigated the mechanism by which tumor cell cycle is arrested by ADSC-CM. We measured the expression of the main proteins involved in cell cycle regulation. We found that ADSC-CM induced a profound inhibition of Rb phosphorylation in Capan-1 cells, with no effect on the protein expression (figure 3A, 3B). Noticeably expression of CDK4 and Cyclin D1, which are critical for Rb phosphorylation, decreased concomitantly. Expression of Cyclin E, which is not involved in the G1-S transition, remained unchanged (Figure 3A; 3B). Of importance, no changes were found in the expression levels of cleaved caspase-3, caspase-8, caspase-9, caspase-6, caspase-7, and PARP following treatment of the cells with ADSC-CM (data not shown). Also, expression levels of cytochrome C, BclXl and Bcl2 remained relatively unchanged in our experimental settings (data not shown).

### ADSCs reduce the *in vivo* growth of human pancreatic tumors

**Figure 4:**
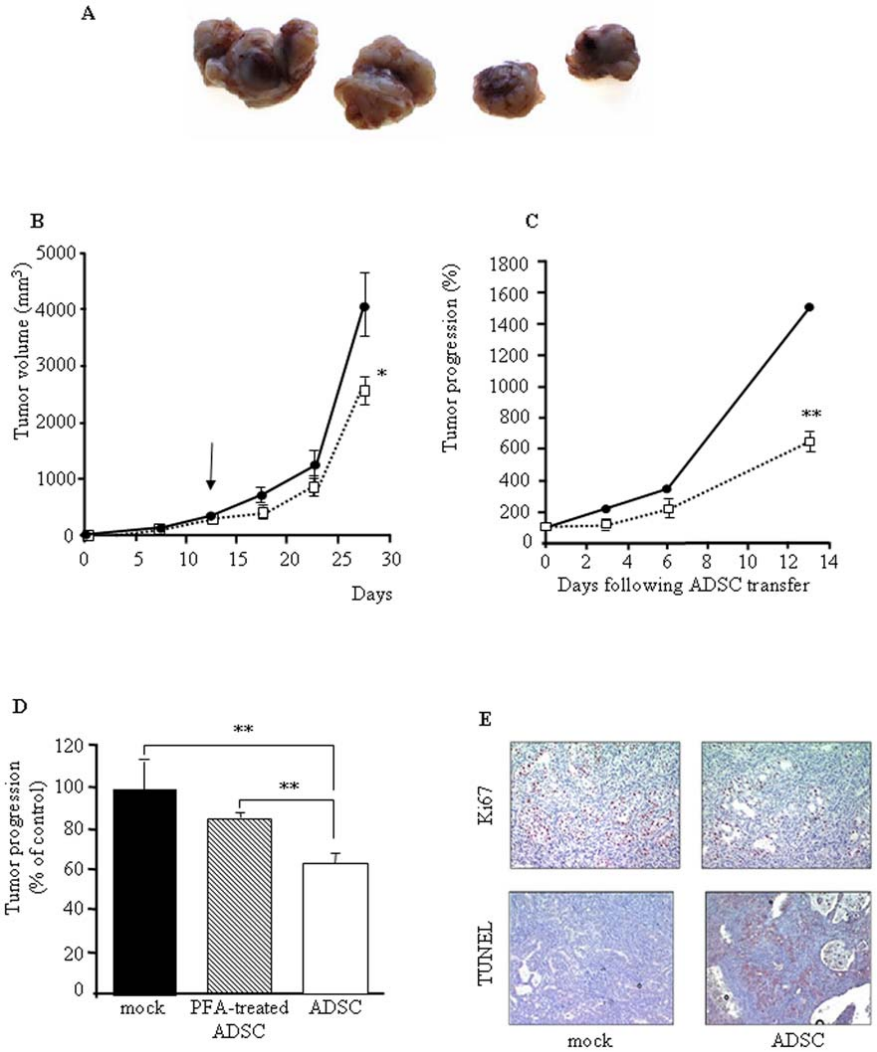
Intratumoral delivery of human ADSCs inhibits pancreatic cancer cell proliferation and induces cell death *in vivo*. Capan-1 tumors were implanted into athymic nude mice (four to six animals per group) as described in Material and Methods. Two weeks later, ADSC were injected within the tumor, and the progression was then monitored. A. Representative resected tumors from athymic nude mice 15 days after receiving no treatment (left) or a single intratumoral dose of 5×10^5^ ADSCs (right). B. The volume of control (black symbols) or ADSC treated (open symbols) tumors was monitored every 2 days, up to 28 days following implantation (13 days following ADSCs injection). C. Percentage of control (black bars) or ADSC treated (open bars) tumor progression was measured at the time indicated following *in vivo* ADSCs injection. D. Tumors were injected either with vehicle, PFA fixed ADSCs or ADSCs and their progression was measured 6 days following intratumoral cell delivery. E. Histological sections of pancreatic tumors injected or not with ADSC were analyzed for proliferation by Ki67 immunostaining (Ki67 labeling index, top panel) or for DNA fragmentation by TUNEL assay (bottom panel), 6 days following ADSC injection. The percentage of labeled nuclei was measured in 15 fields at high magnification (×400) using the visiolab 2000 analysis system. All results were representative of 3 to 4 independent experiments (3 to 5 tumors per group) performed with ADSCs sampled from different healthy donors. Values are means±S.E. *: p<0.05; **: p<0.01; ***: p<0.01.

We next extended ADSCs antiproliferative activities to the inhibition of pancreatic tumors, *in vivo*. Thus, human PDAC tumors were established in athymic mice following the subcutaneous injection of Capan-1 cells as a very aggressive model of PDAC [42], [43]. Fourteen to seventeen days following tumor engraftment, a single dose of ADSCs, corresponding to 10^3^ ADSCs/mm^3^ of tumor was transferred *in vivo* into growing tumors. Control tumors were injected either with vehicle or with paraformaldehyde (PFA) fixed ADSCs. Control tumors grew rapidly, averaging 4076±556 mm^3^ in size by 28 days following implantation (figure 4A, 4B). In contrast, growth of Capan-1 tumors receiving ADSC injection was strongly inhibited (2556±232 mm^3^; figure 4A, 4B). Inhibition of pancreatic tumor growth progression reached −58%±8.9% (p<0.001), two weeks after a single intratumoral injection of ADSCs (figure 4C). Tumor weight was also significantly reduced after ADSCs implantation (control: 4,6 g±0.8 vs ADSCs-treated 2.2 g±0.2, p<0.05). As shown in figure 4D, growth of tumors injected with either vehicle or PFA-treated ADSCs were comparable, showing that the inhibition of tumor growth was not due to an unspecific effect of tumor dilacerations and dependent on ADSC secretion, respectively.

We next investigated the mechanisms involved in the effects of human ADSCs on PDAC tumors *in vivo*. Tumors treated or not with ADSCs were processed for immunohistochemistry for the nuclear antigen Ki67 in order to detect proliferating cells (figure 4E, top panel), or processed for TUNEL analysis to quantify dead cells (figure 4E, bottom panel), 6 days following injection. Quantification of immunostaining demonstrated a significant decrease in the number of proliferating cells, in ADSCs-transferred tumors when compared to control (−66.5%±3.8%; p<0.01). In addition, ADSCs induced a 5-fold increase in cell death in pancreatic tumors as measured by TUNEL assay.

## Discussion

We demonstrated in the present study that human ADSCs sampled from a large panel of different healthy donors efficiently impaired the growth of a very aggressive cancer, i.e. PDAC, both *in vitro* and *in vivo* by inhibiting cancer cell proliferation and promoting cancer cell death following G_1_-phase block. The latter inhibitory effect was extended to malignant cell lines from diverse origins.

Several of our *in vitro* observations reported here demonstrate that ADSCs have the ability to interfere with the proliferation of tumor cells by altering cell cycle progression. The *in vitro* molecular data showed that tumor cells that had been in contact with ADSC-CM were in a resting state (G_0_/G_1_), and down regulated CDK4 and cyclinD1. Interestingly, we were unable to identify either extrinsic or intrinsic-mediated cell death by apoptosis following exposure of cancer cells to ADSCs conditioned medium. This ability to interfere with cell cycle has already been described for stromal cells from other origins [44], [45]. Abnormalities of G_1_-S transition regulators, and more specifically of the Rb pathway, have been recognized as significant factors in the development of human cancers. Consequently, modulation of CDK is a major target for tumor prevention and therapy that may explain why ADSCs inhibit the proliferation of cells derived from multiple epithelial cancers.

The use of ADSC as vehicle in cancer gene therapy has been recently proposed [36], [46], [47]. Indeed ADSCs engineered to convert anticancerous prodrug may be efficient vehicles to potently inhibit the growth of xenografted human colon or prostate tumors [36], [46]. However, only a few studies showing conflicting results have been conducted with naïve cells isolated from adipose tissue [36]–[38], [48]. These discrepancies may be explained by the process of ADSC isolation and passage, the number of injected cells and the procedures used for *in vivo* or *in vitro* studies. Indeed, in some studies, ADSC were injected *in vivo* together with the cancer cells, it is thus difficult to compare the effects of ADSC on installed tumor (in this study) versus growing tumor [36]–[38]. In addition, some data were obtained with mouse cells [38] whereas our model is made of human cells, and cancer cells from diverse origins were used, although we demonstrated that ADSC-CM did not affect tumor cell viability in the same extent. Last and not least, the nature of ADSC varies according to the different studies, since in most cases passaged cells were used [36]–[38], [48] whereas we performed experiments with primary cultures. This may be of importance as ADSC phenotype and potentially function are modified with passages [49]. This variability in the potential effects of stromal cells on cancer cell proliferation has also been described in studies using bone marrow mesenchymal stem cells [16]–[18], suggesting that different mechanisms may be involved according to several factors that remain to be identified. For example, we demonstrate here that cell to cell contact is not fully necessary as anti-proliferative effects were observed both in direct co-culture studies using transwell (no cell contact) and in experiments using ADSCs conditioned medium. In contrast, Khakoo *et al* recently demonstrated that BM-MSCs exerted a potent antioncogenic effect on Kaposi's sarcoma through cell to cell contact and Akt inactivation [18]. This was later on confirmed *in vitro* by Ramasamy *et al* [45].

The identification of the mechanisms involved in stromal and cancer cells interactions and especially the secreted factor responsible for the anti-proliferative effect of ADSC is currently under investigation. Many candidates secreted by ADSCs (such as TGF β1, SDF1, RANTES), interleukines (such as IL6, IL1 β, IL8, GSF, IL11) or prostaglandins (PGE2, PGI2, PGJ2) are known to exert an anti-proliferative/pro-apoptotic effect. Migratory studies performed with Capan revealed that ADSC-CM present no chemoattractant activity (Supplementary Figure S2, and text S1), suggesting that the factor involved in ADSC anti-proliferative effect is not a chemokine. Recently, PGs have been demonstrated to mediate ADSCs mixed lymphocyte reaction inhibition [35]. During the preparation of this manuscript, a study proposed that DKK-1 may be one of these factors (48). IL-6 has been recently involved in protecting normal and premalignant epithelial cells from apoptosis in colitis associated cancer (50). This suggests that this interleukin may play opposite roles depending upon the inflammatory status, the tumor stage and/or type.

*In vivo*, we obtained a yet effective, but transient anti-proliferative effect on tumor progression. GFP-labeled ADSCs were detected surrounding peripheral tumor vessels and within central and peripheral acellular and necrotic areas of the tumor, up to 5 days following injection (Supplementary Figure S3 and Text S1). Based on this observation, the concentration and/or the half-life of the soluble factors responsible for the anti-proliferative/anti-tumoral effect are probably not sufficient to maintain tumor cells in a quiescent state for a long time, but are adequate to initially antagonize cancer cell proliferation. Such transitory effect may be rescued by multiple, intratumoral injection of ADSCs. Delivering human cells into a mouse xenografted with human cancer cells might result in a non-specific xenogenic immune response within the recipient to counteract *in vivo* tumorigenesis. However, because (i) Capan-1 cells do not express MHC from class I and II (data not shown), and (ii) athymic mice immune system (only mediated by NK cells) is less important than in immune animals, we reasonably exclude a non-specific immune response in the observed effect. Considering the ability of ADSC to participate in vascular-like structure formation [23], we cannot rule out a specific pro-angiogenic effect of ADSC in the context of pancreatic tumor growth. However, this effect, if any, seems to be minor at the stage of the disease, i.e. during the exponential phase of tumor growth as we tested in this work. In addition, we were unable to identify changes in vascular density, either from mouse or human origin, following intratumoral injection of ADSCs (data not shown). Alternative changes in ADSCs phenotype following intratumoral injection should be carefully monitored. Indeed, we recently showed that ADSCs rapidly and massively acquired high phagocytic activity and index, when injected into the peritoneal cavity of nude mice [51].

Altogether, our work represents the first study using non-engineered cells to treat PDAC. This anti-proliferative effect of ADSC on pancreatic cancer cells is mediated at least in part by a secreted factor but it is also tempting to speculate that *in vivo* ADSC may modify the microenvironment of the tumor and thus inhibit its proliferation. In conclusion, ADSCs ability to block G_1_-S phase transition and subsequently to inhibit of cancer cells proliferation *in vitro* and *in vivo* may be an efficient and feasible approach for the treatment of the 85% of PDAC patients that cannot be operated in a curative way due to a locally advanced disease.

## Materials and Methods

### Ethic Statement

Human adipose tissue was obtained as waste from aesthetic surgery procedures. All patients gave their written consent. The procedures were approved by the regional ethic committee “Comité de protection des personnes Sud Ouest et Outre Mer I”.

### Chemicals

For adipose tissue digestion, collagenase was purchased from Roche (Roche Diagnostic, Germany). Bovine serum albumin (BSA), dexamethasone, ascorbate-2 phosphate, pantothenic acid, and insulin-transferrin-sodium selenite supplement were purchased from Sigma Aldrich (St. Quentin Fallavier, France). Foetal calf serum (FCS), trypsin, DMEM, RPMI-1640, fungizone, penicillin, streptomycin, and PBS were provided by Invitrogen (Cergy-Pontoise, France). Foetal bovine serum (FBS) for ADSC culture was purchased from Perbio (Paris, France). The antimycoplasma agent, Plasmocin™ was provided by InvivoGen (Toulouse, France).

### Tumor cell culture

Capan-1, Capan-2, BxPC-3, Miapaca-2 and Panc-1 cell lines derived from human PDAC were cultured as described before [52]. HuH7 and HepG2 cells, derived from hepatocellular carcinoma, were cultured as described elsewhere [53]. PC3 (human prostate cancer) and Caco-2 cells (human colon carcinoma) were cultured in DMEM 1 g/l glucose medium supplemented with 10% FCS. Hela (human cervical carcinoma), were cultured in DMEM 4,5 g/l glucose medium supplemented with 10% FCS. All media were supplemented with fungizone, antibiotics, L-Glutamine, and antimycoplasma reagent (Plasmocin™, InvivoGen). Cells were kept at 37°C and 5% CO2.

### Human ADSCs preparation and culture

Human ADSCs were sampled from 20 healthy donors. Stroma vascular fraction was isolated from human adipose tissue obtained from abdominal dermolipectomy in the plastic surgery department of Rangueil Hospital (Toulouse, France) as previously described [19]. Briefly, SVF cells were plated in T75 flasks overnight in DMEM-F12 (1∶1) medium supplemented with 5% FBS, 100 µg/ml pantothenic acid, 100 µM ascorbic acid, 16 µM biotin, 250 µg/ml amphotericin, 5 µg/ml streptomycin and 5 U/ml penicillin. After 24 h, cultures were washed extensively using PBS to remove residual non-adherent cells. HADSC growth was pursued until cells reached 75% confluence (passage 0).

### *In vitro* cell number assessment

50×10^3^ Capan-1 cells were cultured in complete RPMI medium for 24 h in 35-mm dishes, before serum starvation for 16 hours. Cells were cultured in triplicate in the presence or not of ADSCs or BM-MSCs conditioned medium for another 48 hours. Control cells were cultured in DMEM-F12 (1∶1) medium supplemented with 5% FBS. Cell growth was measured by cell counting using a Coulter counter model ZM. For co-culture assays, Capan-1 cells were first plated in cell culture inserts with 4 µm pores (BD biosciences) in complete medium for 24 h. After a 16 h deprivation step, Capan-1 cells were transferred into 35-mm dishes in the presence of ADSCs at different ratios. All experiments were conducted with ADSCs and MSCs purified from different healthy donors.

### *In vitro* cell viability assay

Target cells (15×10^3^) were cultured in complete medium in flat-bottom 96-well plates for 24 h, before serum starvation for 16 hours. Cells were cultured in triplicate in the presence or not of ADSC-conditioned medium for another 48 hours. Control cells were cultured in DMEM-F12 (1∶1) medium supplemented with 5% FBS. Cell viability was measured by MTS assay, according to the manufacturer recommendations (CellTiter96 AQueous Assay, Promega France, Charbonnieres, France). Experiments were conducted with ADSCs purified from different healthy donors. Results are expressed as percentage of values obtained in control conditions.

### Flow cytometry analysis of proliferation and cell death

Capan-1 cells were cultured and treated with ADSCs-CM as described in *“in vitro cell number assessment”* section. Cells were incubated with BrdU 10 µM (Sigma Aldrich) for 4 h at 37°C. Capan were collected, washed once in PBS then fixed in ice-cold 70% ethanol 1 h at 4°C. Cells were collected by centrifugation at 600 g, washed in PBS containing BSA 0.5% and Tween-20 0.5%, and resuspended in HCl 2N and incubated for 30 min at room temperature. After washing, cells were suspended in sodium borate buffer (0.1 M, pH 9) to neutralize residual acid. Cells were stained with anti-BrdU monoclonal antibody (Becton Dickinson) for 30 min at room temperature. After washings, Capan-1 cells were resuspended in propidium Iodide staining solution (5 µg/ml) before analysis on flow cyotmeter (CantoII, Becton Dickinson). For annexin-V and propidium iodide detection, Cells were labelled with either annexin-FITC (Apoptotest FITC, DakoCytomation), or propidium iodide (Invitrogen) as per the manufacturer's recommendation. Apoptotic and necrotic cells were quantified using a BD FACS Calibur (Beckton Dickinson) and Cell quest pro software (Beckton Dickinson).

### *TUNEL* assays

Detection of cell death by TUNEL assay on Capan-1 tumors was done as described elsewhere [42]. For *In vitro* cell-death assays, 50×10^3^ Capan-1 cells were seeded in in 4-well glass slides (Lab-Tek II chamber slide, Nalge Nunc International, Naperville IL) in complete medium for 24 h. After serum starvation for 16 hours, cells were cultured in triplicate in the presence or not of ADSC-CM for another 48 hours. Control cells were cultured in DMEM-F12 (1∶1) medium supplemented with 5% FBS. Detection of the early stages of the chromosome breakdown was performed using Apopdetek, following manufacturer recommendations (ApopDETEK, Enzo life sciences, Farmingdale, NY, USA).

### Western Blot analysis

Proteins were extracted from Capan-1 cells, resolved on SDS-polyacrylamide gels, and transferred to nitrocellulose membrane as described elsewhere [53]. After room-temperature blocking for 1 h, blots were incubated with antibodies purchased from Santa Cruz Biotechnology, Inc (Heidelberg, Germany) raised against cyclinD1 (sc-124), CDK4 (sc-260), CyclinE (sc-247), βactin (sc- 8432) or glyceraldehydes-3-phosphate dehydrogenase (GAPDH) (sc-24778). Rb and phosphospecific Rb antibodies were from Cell signaling Technology (Ozyme, St Quentin en Yvelines, France). Secondary HRP-conjugated antibodies (Perbio Science, Erembogdegem-Aalist, Belgium) were added, and blots were incubated for 1 h at room temperature. Immunoreactive proteins were visualized using ECL immunodetection system (Immobilon, Millipore Corporation, Billerica, MA 01821, USA).

### *In vivo* model of pancreatic cancer

The *in vivo* model of xenografted PDAC-derived cells into athymic mice has been extensively described elsewhere [42]. Briefly, 5×10^6^ exponentially growing Capan-1 cells (100 µl PBS) were inoculated subcutaneously (s.c.) into 8-week-old athymic female nude mice (Swiss nude/nude; IFFA-CREDO, l'Abresle, France) bred and maintained in pathogen-free conditions. On days 14 and 17 following xenograft, intratumoral *in vivo* cell transfer of 10^3^ ADSCs/mm^3^ of tumor was performed with a sterile, 29G insulin needle. As a control, tumors were injected with vehicle or paraformaldehyde (PFA)- treated cells. Each experimental group comprised three to five animals. Four sets of transfer experiments were conducted, with ADSCs sampled from different healthy donors. Tumor size was monitored up to 28 days following transfer as reported elsewhere [42]. At the time of experiment completion, mice were sacrificed; tumors were sampled and frozen in liquid nitrogen or fixed in formalin for further immunohistochemical studies

### Immunostaining for Ki67 in pancreatic tumors

Capan-1 tumors were harvested and fixed in formalin. Four-micrometer-thick sections were prepared from paraffin-embedded sections and rehydrated as described elsewhere [42]. Following antigen retrieval, sections were incubated for 10 min in Protein Block, Serum-free reagent (DakoCytomation) to reduce background staining. Slides were next incubated overnight at 4°C with anti-Ki67 antibody, (1∶100 Clone MIB-1, DakoCytomation). Antibody incubations were done in Dako Antibody diluent (DakoCytomation). After 3 washes in PBS, slides were incubated in 3% H_2_O_2_ for 30 min at room temperature to inhibit endogenous peroxydase. Slides stained for Ki67 were quickly rinsed in distilled water, before 2 washes in PBS, and incubated for 30 minutes at room temperature with biotinylated rabbit anti-mouse IgG (DakoCytomation) and HRP-conjugated streptavidin ABC complex (DakoCytomation) following the manufacturer's instructions. Immunolabeling was visualized with AEC detection reagent (DakoCytomation). After washing in distilled water, slides were counterstained with Mayer's hematoxylin. Resulting immunostaining was observed with an optical microscope, and quantified using VisioLab2000 image analyzer (Biocom, France). For each group, 15 fields were analyzed.

### Statistical analysis

Results are expressed as mean±standard error (SE). One way Anova test or unpaired *t*-test were calculated using Graphpad Instat software, and used to compare data (*: p<0.05, **: p<0.01; ***:p<0.001). p<0.05 was considered significant.

## Supporting Information

Text S1 (0.04 MB DOC)

Figure S1 Flow cytometry analysis of ADSC phenotype. ADSC phenotype was analyzed by flow cytometry using a combination of cell surface markers. Dot plots representative of 6–10 different cell cultures showed homogeneous population positive for stromal cell markers (CD34, CD90, HLA-ABC, CD73) and negative for hematopoietic (CD45) and endothelial (CD31) cell surface markers.
(0.08 MB TIF)

Figure S2 ADSC conditioned media present no chemoattractant activity on Capan-1 cells. Chemotactic migration of Capan-1 cells was performed as described in Suplementary methods, with three different ADSC-conditioned media. A. Quantification of chemotaxis (chemotactic index) is calculated by chemotaxis/spontaneous migration. Values are means±S.E. of three separate experiments. B. Pattern of migration in response to control medium. C. Pattern of migration in response to ADSC conditioned medium. B and C are representative of three independent expriments.magnifications ×4.
(0.25 MB TIF)

Figure S3 GFP-labeled ADSC are readily detected in pancreatic tumors following intratumoral injection. Capan-1 tumors were implanted into athymic nude mice as described in Materials and Methods. hADSC transduced with a lentiviral vector encoding for GFP were injected intratumorally. 5 days later, tumors were sampled, fixed in paraformaldehyde and analyzed for GFP expression. GFP expression was detected by immunochemistry as described in Suplementary Methods in control-injected (A) and ADSC-injected samples (B, C). Results are representative of three tumors injected with GFP- labeled hADSC. magnification ×400.
(0.31 MB TIF)
