# Supplementary material for: Adult Stromal Cells Derived from Human Adipose Tissue Provoke Pancreatic Cancer Cell Death both In Vitro and In Vivo
Source: PLoS One. 2009 Jul 17;4(7):e6278. doi: 10.1371/journal.pone.0006278 (PMC2707007; doi:10.1371/journal.pone.0006278)
Supplement: Text S1 — (0.04 MB DOC) [file pone.0006278.s001.doc]

**SUPPLEMENTARY TEXT S1**

**Supplementary Methods**

**Cell phenotyping**. Isolated cells were analyzed by flow cytometry (FACS) using the following protocol. Human ADSC (P0) were stained in staining buffer (SB) consisting of phosphate-buffered saline containing 0.5% new calf serum and FcR Block reagent (StemCell Technologies, Vancouver). Cells were incubated with anti-mouse monoclonal antibodies (mAb) or rat immunoglobulins (isotypes) conjugated with Fluorescein Isothiocyanate (FITC), Phycoerythrin (PE), Peridinin Chlorophyll Protein (PerCP) or Allophycocyanin (APC) for 30 min at 4°C. Triple or quadruple staining was performed by incubating cells with CD31, CD90, HLA ABC, CD73, CD45 and CD34 conjugated primary antibodies in one step, in order to precisely characterize the cell population. Cells were washed in SB and then analyzed on a fluorescence-activated cell sorter (FACS) (FACS CantoII, Becton Dickinson, Mountain View, CA). Data acquisition and analysis were then performed (Diva software, Becton Dickinson). All antibodies and their isotype controls were purchased from Becton Dickinson.

**Under-agarose cell migration assay.** A modified version of the under-agarose assay was used to study chemotactic migration of Capan-1 (Ramjeesingh et al., 2003). In brief, a 2% (w/v) agarose solution in KR buffer was mixed with fetal calf serum (FCS). This agarose solution was then placed in 60mm diameter plates, and allowed to solidify overnight at 4°C. Eight holes were punctured at equal distance apart in the agarose sheet, by using a hole puncher. Capan-1 cells were collected and resuspended in MEMα 2% plasma, at a density of 10000 cells/µl. Capan cells (500000 in 5µl) were placed in the internal hole. Different ADSC conditioned media or control medium were placed in the external holes. Cells were allowed to migrate under the agarose sheet for 4h at 37°C and then fixed with methanol for 30min and then formaldehyde (37%) for 30min at room temperature. Plates were stained with Giemsa 6% for 20min at room temperature and then washed twice with PBS, before analysis under optical microscope.

**Vector production and hADSC transduction.** Vector particles were produced by transient calcium phosphate co-transfection of 293 FT cells with the vector plasmid carrying EGFP, pCMVΔ8.91 and pHCMV-G as previously described (Sirven et al., 2000). Vector concentrations were determined by measuring p24. The viral titres were evaluated on the 293FT cells and expressed in TU/ml (Transduction Unit/ml). For transduction, hADSC were plated at 4.10^3^ cells per cm^2^. At subconfluence, lentiviral particles were added at 2.8.10^6^ TU/ml corresponding to MOI (multiplicity of infection) 2. After 48h of transduction, cells were washed twice with PBS 1X and cultured in the same conditions for another 72h. EGFP expression was determined by flow cytometry.

**Immunostaining for GFP in pancreatic tumors.** Capan-1 tumors were harvested and fixed in formalin. Four-micrometer-thick sections were prepared from paraffin-embedded sections. Sections were deparaffinized in xylene, and rehydrated in descending grades (100 to 50%) of ethanol with a final wash in TBS. For GFP detection, sections were incubated in 10 mM citrate buffer (pH 6.0) at 97°C for 45 min. After 3 washes in PBS (pH 7.4), sections were incubated for 10 min in Protein Block, Serum-free reagent (DakoCytomation) to reduce background staining. When detecting GFP, slides were incubated in 3% horse serum diluted in TBS for 20 min. Slides were next incubated for 1h with rabbit anti-GFP antibody (1:1000, Abcam), or overnight at 4°C. Antibody incubations were done in Dako Antibody diluent (DakoCytomation). After 3 washes in TBS, slides were incubated in 3% H_2_O_2_ for 30 min at room temperature to inhibit endogenous peroxydase. GFP was detected using ImmunoImpress kit (vector), following the manufacturer’s instructions. Immunolabeling was visualized using 3,3'-diaminobenzidine solution (Sigma Co. St. Louis, MO, USA). After washing in distilled water, slides were counterstained with Mayer's hematoxylin. Resulting immunostaining was observed with an optical microscope, and quantified using VisioLab2000 image analyzer (Biocom, France). For each group, 15 fields were analyzed.

**References**

- Ramjeesingh R., Leung R and Siu CH. Interleukin-8 secreted by endothelial cells induces chemotaxix of melanoma cells through the chemokine receptor CXCR1. (2003) FASEB J. 17(10):1292-4.

- Sirven A, Pflumio F, Zennou V, Titeux M, Vainchenker W, Coulombel L, Dubart-Kupperschmitt A, Charneau P. The human immunodeficiency virus type-1 central DNA flap is a crucial determinant for lentiviral vector nuclear import and gene transduction of human hematopoietic stem cells. (2000) Blood 96:4103-10.

**Supplementary Figure Legends**

**Supplementary Figure S1: Flow cytometry analysis of ADSC phenotype.** ADSC phenotype was analyzed by flow cytometry using a combination of cell surface markers. Dot plots representative of 6-10 different cell cultures showed homogeneous population positive for stromal cell markers (CD34, CD90, HLA-ABC, CD73) and negative for hematopoietic (CD45) and endothelial (CD31) cell surface markers.

**Supplementary Figure S2**: **ADSC conditioned media present no chemoattractant activity on Capan-1 cells.** Chemotactic migration of Capan-1 cells was performed as described in Suplementary methods, with three different ADSC-conditioned media. **A.** Quantification of chemotaxis (chemotactic index) is calculated by chemotaxis/spontaneous migration. Values are means ± S.E. of three separate experiments. **B.** Pattern of migration in response to control medium. **C.** Pattern of migration in response to ADSC conditioned medium. **B** and **C** are representative of three independent expriments. magnifications x4.

**Supplementary Figure S3: GFP-labeled ADSC are readily detected in pancreatic tumors following intra-tumoral injection**. Capan-1 tumors were implanted into athymic nude mice as described in Materials and Methods. hADSC transduced with a lentiviral vector encoding for GFP were injected intratumorally. 5 days later, tumors were sampled, fixed in paraformaldehyde and analyzed for GFP expression. GFP expression was detected by immunochemistry as described in Suplementary Methods in control-injected (A) and ADSC-injected samples (B, C). Results are representative of three tumors injected with GFP- labeled hADSC. magnification x400.
