# Supplementary figures and images for: Adult Stromal Cells Derived from Human Adipose Tissue Provoke Pancreatic Cancer Cell Death both In Vitro and In Vivo
Source: PLoS One. 2009 Jul 17;4(7):e6278. doi: 10.1371/journal.pone.0006278 (PMC2707007; doi:10.1371/journal.pone.0006278)

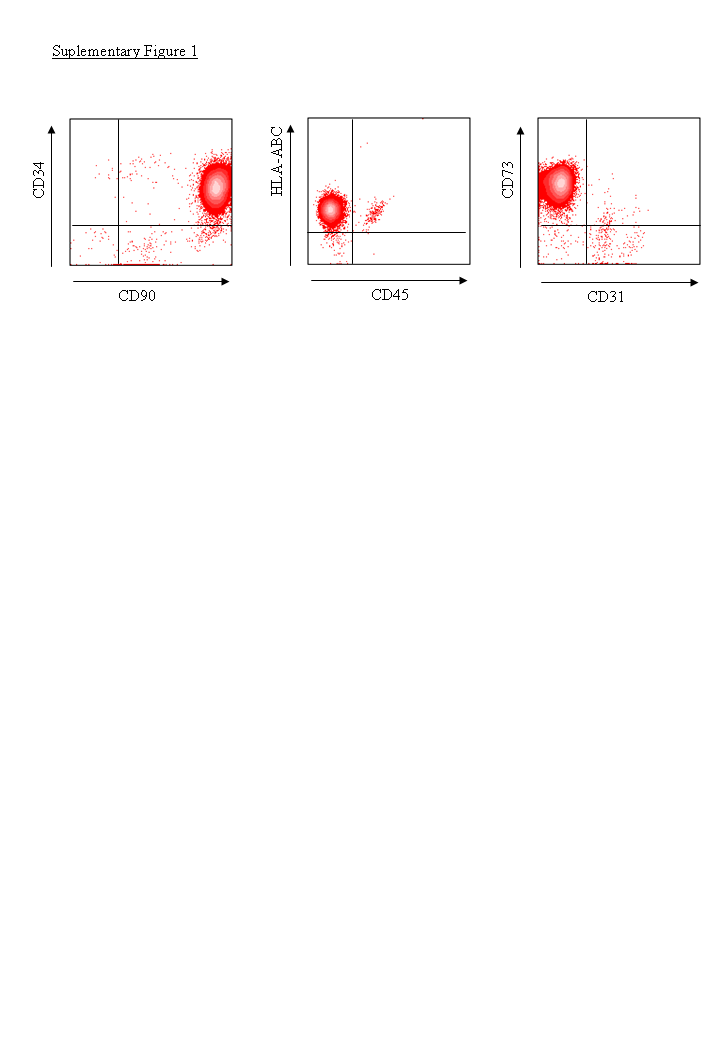

Supplement: Figure S1 — Flow cytometry analysis of ADSC phenotype. ADSC phenotype was analyzed by flow cytometry using a combination of cell surface markers. Dot plots representative of 6–10 different cell cultures showed homogeneous population positive for stromal cell markers (CD34, CD90, HLA-ABC, CD73) and negative for hematopoietic (CD45) and endothelial (CD31) cell surface markers. (0.08 MB TIF) [file pone.0006278.s002.tif]

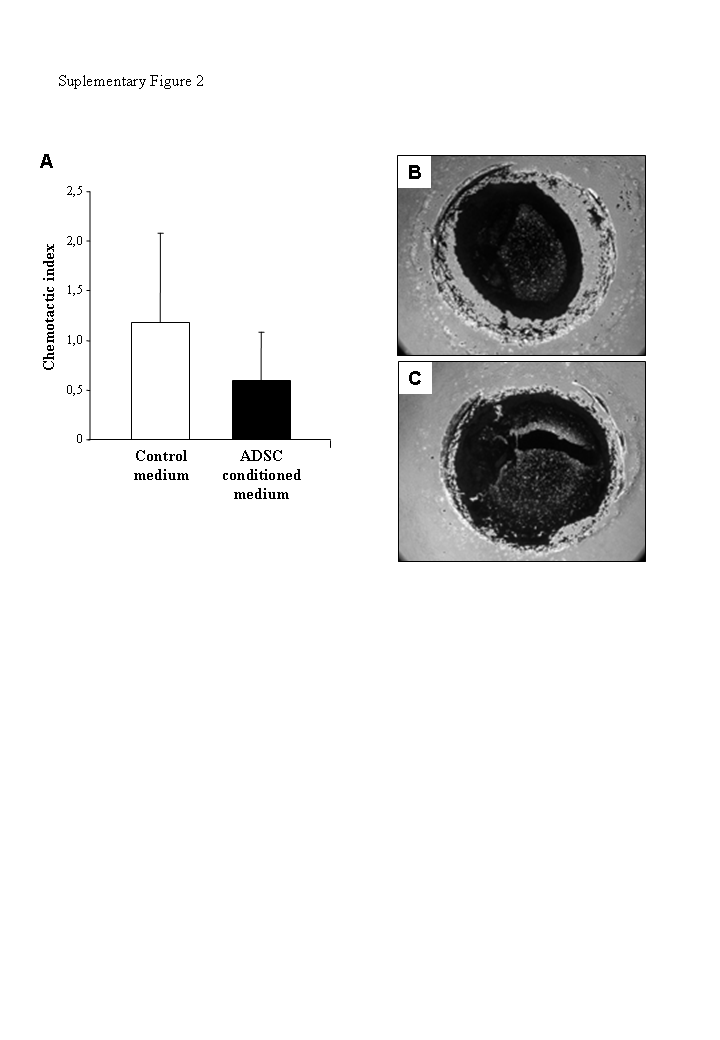

Supplement: Figure S2 — ADSC conditioned media present no chemoattractant activity on Capan-1 cells. Chemotactic migration of Capan-1 cells was performed as described in Suplementary methods, with three different ADSC-conditioned media. A. Quantification of chemotaxis (chemotactic index) is calculated by chemotaxis/spontaneous migration. Values are means±S.E. of three separate experiments. B. Pattern of migration in response to control medium. C. Pattern of migration in response to ADSC conditioned medium. B and C are representative of three independent expriments.magnifications ×4. (0.25 MB TIF) [file pone.0006278.s003.tif]

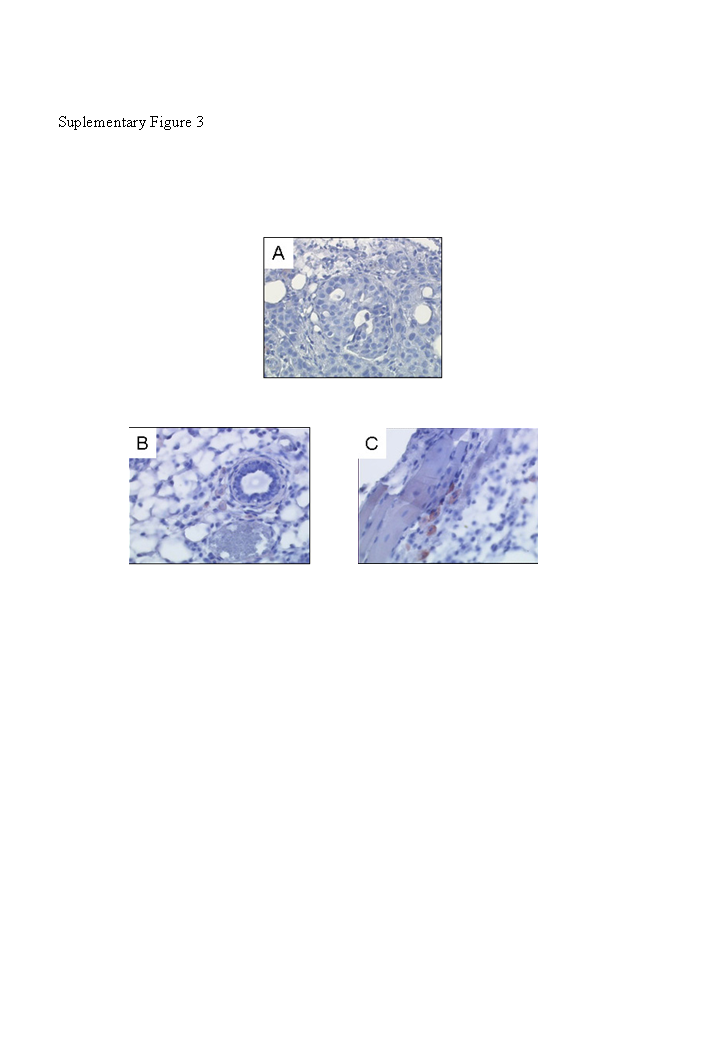

Supplement: Figure S3 — GFP-labeled ADSC are readily detected in pancreatic tumors following intratumoral injection. Capan-1 tumors were implanted into athymic nude mice as described in Materials and Methods. hADSC transduced with a lentiviral vector encoding for GFP were injected intratumorally. 5 days later, tumors were sampled, fixed in paraformaldehyde and analyzed for GFP expression. GFP expression was detected by immunochemistry as described in Suplementary Methods in control-injected (A) and ADSC-injected samples (B, C). Results are representative of three tumors injected with GFP- labeled hADSC. magnification ×400. (0.31 MB TIF) [file pone.0006278.s004.tif]
